# The Scent of Ant Brood: Caste Differences in Surface Hydrocarbons of *Formica exsecta* Pupae

**DOI:** 10.1007/s10886-021-01275-w

**Published:** 2021-04-26

**Authors:** Unni Pulliainen, Nick Bos, Patrizia d’Ettorre, Liselotte Sundström

**Affiliations:** 1 Organismal and Evolutionary Biology Research Programme, Faculty of Biological and Environmental Sciences, University of Helsinki, Helsinki, Finland; 2 Tväminne Zoological Station, Faculty of Biological and Environmental Sciences, University of Helsinki, Helsinki, Finland; 3 Department of Biology, Faculty of Sciences, University of Copenhagen, Copenhagen, Denmark; 4 Laboratory of Experimental and Comparative Ethology, University of Paris, 13, Sorbonne Paris Cité, Paris, France

**Keywords:** Social insects, Ants, Surface hydrocarbons, Caste, Pupae, *Formica*

## Abstract

Chemical communication is common across all organisms. Insects in particular use predominantly chemical stimuli in assessing their environment and recognizing their social counterparts. One of the chemical stimuli used for recognition in social insects, such as ants, is the suite of long-chain, cuticular hydrocarbons. In addition to providing waterproofing, these surface hydrocarbons serve as a signature mixture, which ants can perceive, and use to distinguish between strangers and colony mates, and to determine caste, sex, and reproductive status of another individual. They can be both environmentally and endogenously acquired. The surface chemistry of adult workers has been studied extensively in ants, yet the pupal stage has rarely been considered. Here we characterized the surface chemistry of pupae of *Formica exsecta*, and examine differences among sexes, castes (reproductive vs. worker), and types of sample (developing individual vs. cocoon envelope). We found quantitative and qualitative differences among both castes and types of sample, but male and female reproductives did not differ in their surface chemistry. We also found that the pupal surface chemistry was more complex than that of adult workers in this species. These results improve our understanding of the information on which ants base recognition, and highlights the diversity of surface chemistry in social insects across developmental stages.

**Supplementary Information:**

The online version contains supplementary material available at 10.1007/s10886-021-01275-w.

## Introduction

Communication is essential for maintaining cohesion whenever living related units interact, from cells within organisms to individuals within societies. Chemical information is one of the oldest, and most common modes of communication (d’Ettorre and Moore 2008). The use of chemical cues and signals is particularly widespread in social insects, in which group cohesion and attainment of inclusive fitness benefits strongly rely on the ability to distinguish nest-mates from non-nest-mates, and different classes of nest-mates (Jaisson 1991). Through accurate recognition via chemical cues, intruders are kept out, shared resources are kept within the group, and division of labor is optimized (Martin and Drijfhout 2009b; Pamminger et al. 2014).

Most research on chemical communication in ants has been conducted on adult workers (d’Ettorre and Lenoir 2011; Sturgis and Gordon 2012; Tsutsui 2013; van Zweden and d’Ettorre 2010). A mixture of compounds on the surface of eusocial insects, cuticular hydrocarbons, is responsible for within and between species recognition (Martin and Drijfhout 2009a). Production of surface hydrocarbons is partly genetically controlled (Wicker-Thomas and Chertemps 2010), but surface hydrocarbons may vary across individuals (van Zweden et al. 2010). Thus, the actual blend of surface hydrocarbons is also influenced by the surrounding environment (Martin et al. 2013; van Zweden and d’Ettorre 2010; van Zweden et al. 2010). For instance, in *Polistes* wasps, nest-specific odors are acquired from nest material (Bos et al. 2011; Gamboa 2004; Katzav-Gozansky et al. 2004), from interactions between social insect hosts and their social parasites (Lorenzi 2006), or cues become homogenized through trophallaxis and grooming (Boulay et al. 2000; Leboeuf et al. 2016; Soroker and Hefetz 2000).

Different classes of surface hydrocarbons can convey different signals or be used as cues in different contexts. Of the major classes of hydrocarbons found in ants, the *n*-alkanes are structurally optimal for waterproofing (Gibbs 1998), which is one of the original functions of these hydrocarbons. In some species, the amount and proportion of *n-*alkanes are environmentally determined (Dani et al. 2005; Martin et al. 2008c; van Zweden et al. 2009), and have been shown to vary between tasks in the ant *Formica exsecta* (Martin and Drijfhout 2009b), as well as other ants (e.g., Wagner et al. 1998). The information content of hydrocarbons increases with the addition of double bonds (alkenes), or methyl-branches (Guerrieri et al. 2009; Lorenzi et al. 2011; Martin et al. 2008b). Indeed the (Z)-9-alkenes have a significant role as nest-mate recognition cues for adult workers (Martin et al. 2008c, 2013) in *F exsecta*, whereas C_25_ dimethyl-alkanes have a similar role in *F. fusca* (Martin et al. 2008a). Ants are able to detect and react to *n-*alkanes (Bos et al. 2012), alkenes (Martin et al. 2008c), and methylated alkanes (Guerrieri et al. 2009). Hence, variation within each class of hydrocarbons could potentially be used by the ants as source of information.

Differences in the qualitative composition of hydrocarbons in a cuticular profile can be used particularly in interspecific recognition (Martin et al. 2008b), whereas within species the hydrocarbon profiles usually comprise the same set of hydrocarbons, which can vary quantitatively between colonies, but also according to age classes, castes, tasks, fecundity, and/or gender (e.g., Dietemann et al. 2005; Kleeberg et al. 2017; Martin et al. 2008c; Martin and Drijfhout 2009b). Thus, to discriminate among different categories of colony members (e.g., gender or developmental stage), individual ants must be able to discriminate not only between different hydrocarbons, but also between different concentrations and ratios (Martin et al. 2008a; di Mauro et al. 2015).

Chemical communication in brood has received much less attention than that among adult individuals of social insects, yet surface chemicals play a key role also in brood recognition (Achenbach et al. 2010; Helanterä and d’Ettorre 2014; Souza et al. 2006; Viana et al. 2001). Recognizing the identity of brood is crucial for targeting brood care to the correct individuals, as well as discriminating according to caste, maternity, sex, or developmental stage (Schultner et al. 2017). Brood discrimination according to colony and species may also be important for maintaining colony integrity against the intrusion by social parasites (Lenoir et al. 2001; Schmid-Hempel 1998). Brood surface chemistry has been shown to differ among species, populations, colonies (Achenbach and Foitzik 2009; Achenbach et al. 2010; Brian 1975; Helanterä and d’Ettorre 2014; Johnson et al. 2004; Richard et al. 2007; Schultner et al. 2013; Souza et al. 2006; Viana et al. 2001), castes (Achenbach et al. 2010; Brian 1975; Penick and Liebig 2017; Villalta et al. 2016), according to viability (Dietemann et al. 2005), developmental stage (Johnson et al. 2004; Richard et al. 2007), gender (Achenbach et al. 2010), maternity (Endler et al. 2006; Helanterä and d’Ettorre 2014; Meunier et al. 2010), and the social structure of the colony (Meunier et al. 2011).

Here we study the surface chemistry of pupae of the ant *Formica exsecta*. Adult workers of this species have been extensively studied with respect to nest-mate recognition and surface chemistry (e.g. Martin and Drijfhout 2009a; Martin et al. 2008a, 2012a,b, 2013). The adult chemical profile is very simple, consisting of four *n*-alkanes, which relate to task differences (Martin and Drijfhout 2009b), and four (Z)-9-alkenes, which have been found to act as nest-mate recognition cues (Martin et al. 2008c, 2013). The surface chemistry of eggs (Helanterä and d’Ettorre 2014), and larvae (Peignier et al. 2019) of *F. exsecta* have also been studied, but to our knowledge the surface chemistry of pupae remains uncharted. We characterize the surface chemistry of both sexual and worker pupae in this species, how it relates to the chemistry of adult workers, and explore the qualitative and quantitative differences among them. We examine whether different castes, sexes, and parts (cocoon *vs.* developing individual) carry specific chemical cues that would allow distinguishing them from each other. As the cocoon separates the developing individual inside it from the outside world, we hypothesize that key compounds may differ between the two, and that the cocoon contains the nestmate recognition cues due to passive contact with the workers. Finally, we explore the variation in surface chemistry between colonies.

## Methods and Materials

### Data Collection

We collected workers and pupae of *F. exsecta* (Nylander 1846) from 35 colonies in our study population in the Tvärminne archipelago by the Hanko peninsula of southwestern Finland (Table 1). Workers and pupae were brought to the laboratory and placed in nest boxes lined with Fluon ® (Whitford, United Kingdom) to prevent the ants from escaping until further processing. The ants were fed Bhatkar-Whitcomb diet (Bhatkar and Whitcomb 1970), and water was provided daily.

**Table 1:** Samples and terminology

Caste	Sexuals				Workers		
Gender	Gyne pupae		Male pupae		pupae		adults
Sample	cocoons	developing individuals	cocoons	developing individuals	cocoons	developing individuals	
*N* colonies	13	13	15	15	20	20	35
*N* individuals	44	44	56	55	100	97	199

### Chemical Analysis of Surface Hydrocarbons

The caste and gender (worker, male, gyne = young reproductive female) of the pupae was determined based on morphological characteristics (size, the shape of the abdomen, and the eyes). The surface chemistries of pupae and adult workers were then analyzed with gas chromatography coupled with mass spectrometry (GC–MS). For each colony, we used five individuals of each caste: adult workers, worker pupae, and sexual pupae, when only either males or females were available. If both male and female pupae were available we used three males and three females. Each pupa provided two separate samples, one comprising the pupal case (henceforth ‘cocoon’), and one comprising the individual itself (henceforth ‘developing individual’), which were placed in separate glass vials (Pulliainen et al. 2018). The surface chemicals were extracted by submerging each sample in 120 µl pentane (HPLC-grade > 99.9% purity, Sigma–Aldrich, France) for 10 min, which was then allowed to evaporate. The samples were then re-diluted in 50 µl pentane, containing an internal standard (5 ng/µl of *n*-C_18,_ Sigma–Aldrich, France), and analyzed with an Agilent 7890A GC coupled with an Agilent 5975c MS (Agilent Technologies, Santa Clara, CA, U.S.A.) (Pulliainen et al. 2018). A solvent-only control was used to check for contamination in every 10 samples. The compounds were integrated using MSD Chemstation (Agilent). Compounds were identified by their retention time, fragmentation patterns and diagnostic ions, and comparison with published results (Martin et al. 2008a, b, c; Martin and Drijfhout 2009b; Martin et al. 2013). To de-convolute and identify co-eluting peaks, to detect and subtract the background noise from small peaks, and to verify the absence of missing peaks we used the AMDIS 32 software (National Institute of Standards and Technology, Gaithersburg, MD, U.S.A.). We found that the deconvoluted peaks do not necessarily contribute equally to the different principal components (see below), and can even contribute in opposing directions (Supplementary Fig. S1, Pulliainen et al. 2018).

### Statistical Analyses

The samples comprised chemical data from seven groups: (1) gyne cocoons (female reproductives), (2) developing gynes, (3) male cocoons, (4) developing males (5) worker cocoons, (6) developing workers, and (7) adult workers (Table 1). We included all detected compounds in the analysis, which were present at > 1% of the cumulative peak area in at least one individual (Table 2). We standardized the peak areas by calculating the ln(*P*_*i,*_* /g*(*P*)) (Aitchison 1986), where *P*_*i*_ is the area of a peak, and *g(P)* is the geometric mean of all the peak areas of the sample. We then performed a principal component analysis (PCA) on the standardized peak areas, to reduce the number of variables. The first PC explained 54.2%, and the second an additional 13% of the variation. For the analysis we retained seven principal components (PCs), which together explained 85.4% of the total variance (original data in Pulliainen et al. 2018).

**Table 2:**
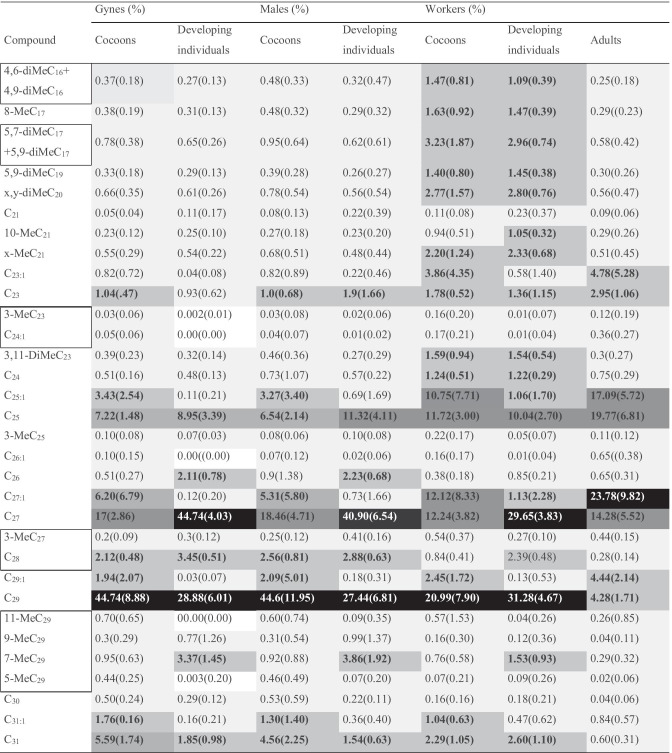
Average percentage of each of the compounds of the chemical profile for each of the groups, with standard deviations given in brackets

Absent compounds are shaded in white. Compounds that were on average over 1% in each category are bolded and shaded with gradually darker colours with increasing percentage. Co-eluted compounds are surrounded with black borders. Details on retention times and identification are given in Table S1

All statistical analyses were done in R, version 3.5.2. (https://www.r-project.org/). To assess whether cocoons, developing individuals, castes, and/or genders differed in their surface chemistry, we used pairwise permutational MANOVA on the PCs, with sample category (e.g. gyne cocoons, male cocoons etc.) as a factor. In the analysis we used both sequential Bonferroni, and Holm-Bonferroni -adjusted P-values, the Euclidean simulation method set to 999 permutations, and the function pairwise.adonis in the R package pairwiseAdonis (Arbizu 2019). Adonis truncates all *P*-values to three decimals and the permutation procedure produces minor random variation in *P*-values, which may be critical in the case of borderline significances. Thus, we ran the analysis 100 times and report the mean and standard deviation for the *P*-values obtained. When *P*-values are below 0.001 there is no variance since all *P*-values are then truncated to 0.001. To verify our results further, we also performed a linear discriminant analysis (LDA) on the PCs for the different sample categories, using the function *lda* in the R package MASS (Venables and Ripley 2002). Finally, given that *n*-alkenes have been shown to carry information on colony identity, whereas other compounds may have other functions (Martin and Drijfhout 2009a) we tested for chemical information on colony identity with a permutational MANOVA on all compounds, on *n*-alkenes only, and on all compounds except *n*-alkenes. In this analysis we did the peak standardizations and PCAs separately for each data set, and used the PCs obtained separately for each sample group (Table 1) as the response variable, and colony as a fixed effect. The permutational MANOVA was set to 1000 permutations, using the Euclidean method, and the Adonis function in the R package vegan (Oksanen et al. 2019).

## Results

### Quantitative Differences

**Fig. 1:**
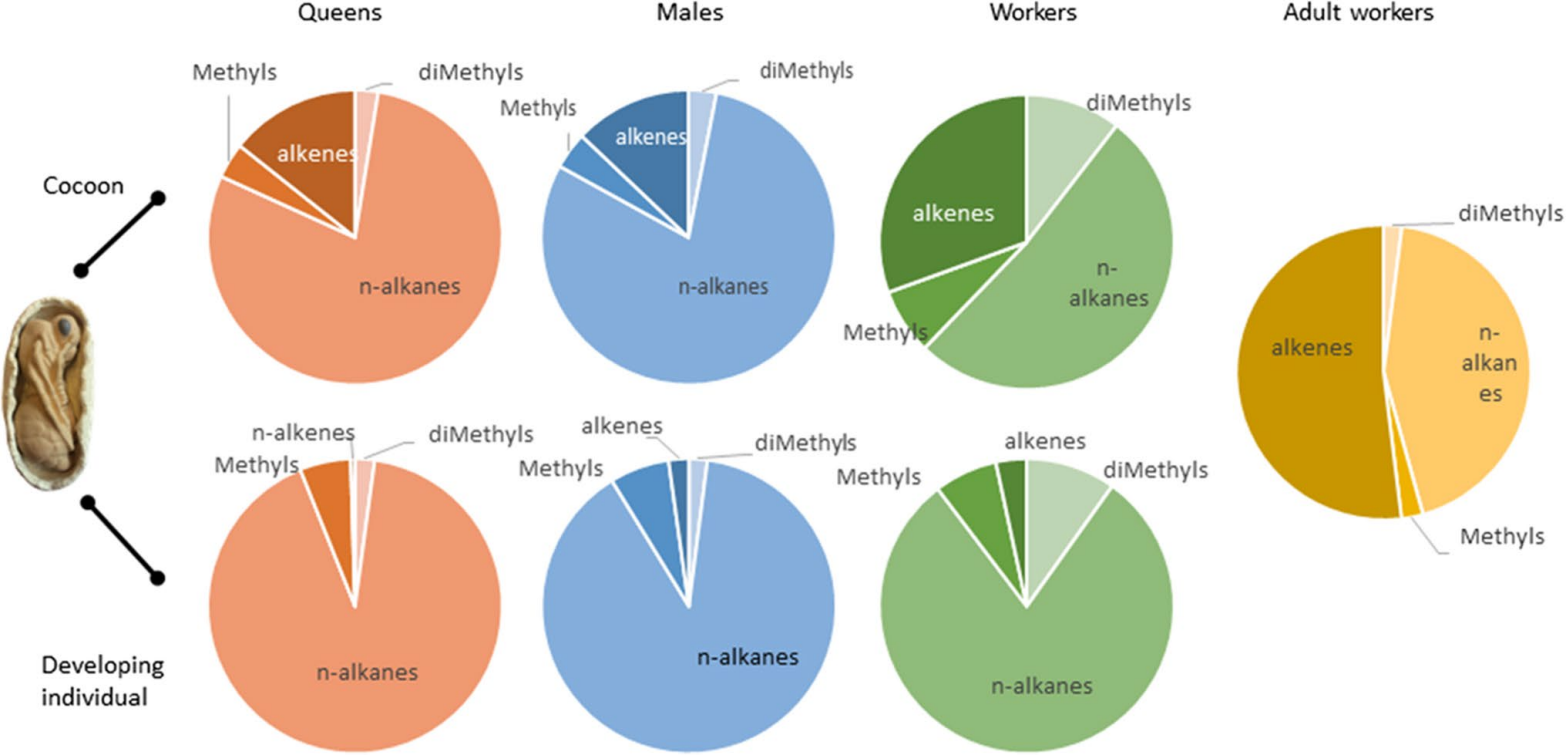
Relative representation of groups of hydrocarbons (*n*-alkanes, alkenes, monomethylated and dimethylated alkanes) in gyne, male, and worker pupae, as well as adult workers. Photo ©Unni Pulliainen

We identified 32 compounds, which comprised alkenes (C_23:1_-C_31:1_), *n-*alkanes (*n-*C_21_- *n-*C_31_), and methyl-branched alkanes (mono-, and dimethyls) (Table 2, Fig. 1). According to the MANOVA the chemical profiles differed significantly among colonies, across all sample categories, and data sets (all compounds, alkenes only, and all compounds but alkenes), except for the data set with all but alkenes in developing gynes (Table 3).

**Table 3:** Results of permutational MANOVA assessing whether the samples carry colony information

	All compounds		Only alkenes			No alkenes		
	*R*^*2*^	*P*	*R*^*2*^		*P*	*R*^*2*^	*P*	*df*
gyne cocoons	0.54	0.003	0.52		< 0.001	0.58	< 0.001	12
gyne dev,individuals	0.45	0.008	0.42		0.004	0.36	0.14	12
male cocoons	0.48	0.002	0.40		0.004	0.53	< 0.001	14
male dev,individuals	0.39	0.014	0.37		0.004	0.41	< 0.001	13
worker cocoons	0.37	0.002	0.34		< 0.001	0.39	< 0.001	19
worker dev,individuals	0.38	< 0.001	0.42		< 0.001	0.37	< 0.001	19
adult workers	0.52	< 0.001	0.43		< 0.001	0.50	< 0.001	35

The surface chemistry of males and gynes (both cocoons and developing individuals), did not differ significantly following the Holm-Bonferroni correction (MANOVA, cocoons: *R*^*2*^ = 0.06, *P* = 0.175; developing individuals: *R*^*2*^ = 0.07, *P* = 0.085, Table 4, Fig. S2a, b in Supplementary Material). In these cases, only 68% and 67% of the samples, respectively, were correctly classified (Table 4). The corresponding differences between worker and sexual samples were statistically significant following the Holm-Bonferroni corrections with identical *P*-values, (MANOVA, worker *vs.* gyne cocoons: *R*^*2*^ = 0.25, *P* = 0.021; worker *vs.* gyne developing individuals: *R*^*2*^ = 0.14, *P* = 0.021, worker *vs.* male cocoons: *R*^*2*^ = 0.12, *P* = 0.021; worker *vs.* male developing individuals: *R*^*2*^ = 0.18, *P* = 0.021, (Table 4; Fig. S2c, d in Supplementary Material). In these cases 82–94% of the samples were correctly classified (Table 4). Within each caste (gynes, males and workers), cocoons differed significantly in their chemical profile from developing individuals, again with identical results following Holm-Bonferroni corrections (MANOVA, gynes: *R*^*2*^ = 0.69, *P* = 0.021, males: *R*^*2*^ = 0.26, *P* = 0.021; workers: *R*^*2*^ = 0.39, *P* = 0.021). In these cases, 90–98% of the samples were correctly classified to category (Table 4).

**Table 4:** Results from pairwise permutational MANOVA with adjusted *P*-values (Holm-Bonferroni correction), and a linear discriminant analysis (LDA)

	Pairs	*Df*		Sum of Squares	*F*.model		*R*^*2*^		P adj (SD)^a^		% correctly assigned to category (LDA)
Individuals	worker vs. gyne	1		310.08	22.97		0.14		0.021 (0) ^b^		90
	worker vs. male	1		544.36	32.94		0.18		0.021 (0)		82
	male vs. gyne	1		105.74	7.06		0.07		0.085 (0.039)		67
Cocoons	worker vs. gyne	1		643.11	48.46		0.25		0.021 (0)		94
	worker vs. male	1		385.26	21.53		0.12		0.021 (0)		87
	male vs. gyne	1		92.68	6.10		0.06		0.175 (0.054)		68
Gynes	cocoon vs. individual	1		1657.24	193.98		0.69		0.021 (0)		98
Males	cocoon vs. individual	1		761.36	37.58		0.26		0.021 (0)		90
Workers	cocoon vs. individual	1		1924.07	123.98		0.39		0.021 (0)		91
	adult vs. individual	1		4136.13	361.57		0.55		0.021 (0)		98
	adult vs. cocoon	1		611.55	52.08		0.15		0.021 (0)		86
	adult vs. gyne cocoon	1		902.26	73.82		0.23		0.021 (0)		98
	adult vs. male cocoon	1		1003.15	109.83		0.31		0.021 (0)		99

^a^average and standard deviation for 100 permutations

^b^Adonis truncates all p-values to three decimals, hence the values < 0.001 are identical and variance equals 0

**Fig. 2:**
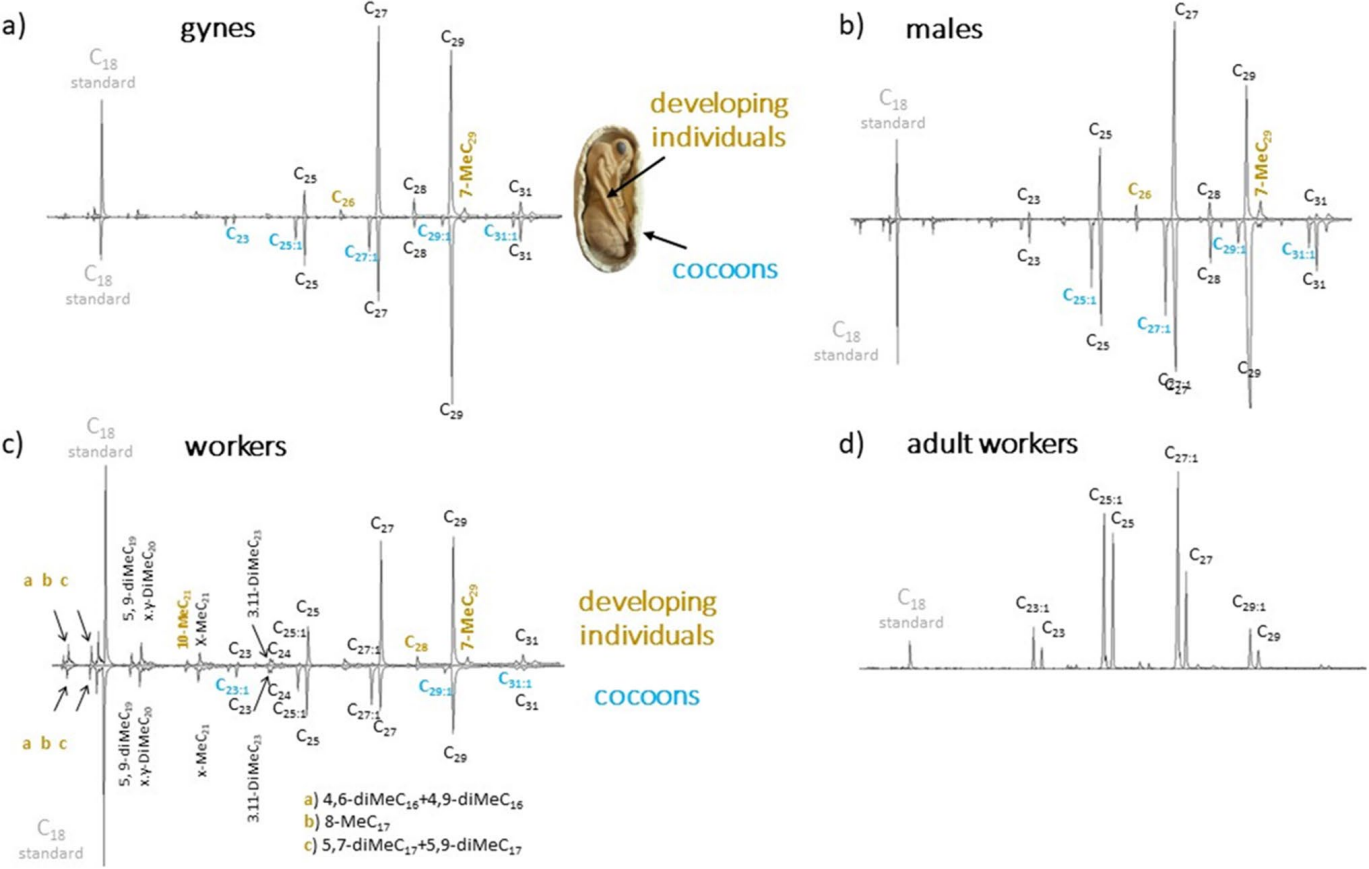
Representative chromatograms of cuticular hydrocarbons in gyne, male and worker pupae (a, b & c), with chromatograms from developing individuals on top and those from cocoons below, as well as a representative adult worker chromatogram (d). Compounds only present in cocoons are indicated in yellow/grey, and those present in developing individuals in blue/grey, within each group (gyne, male or worker pupae, or adult workers). Note scale of y-axis differs in as indicated by size of peak for internal standard (C_18_)

Adult workers also differed in their surface chemistry both from developing worker individuals (MANOVA, *R*^*2*^ = 0.55, *P* = 0.021, Fig. S2g in Supplementary Material), and the cocoons (MANOVA, worker cocoons: *R*^*2*^ = 0.15, *P* = 0.021, male cocoons: *R*^*2*^ = 0.23, *P* = 0.021, gyne cocoons: *R*^*2*^ = 0.31, *P* = 0.021). In these cases 86–99% of the samples were correctly classified (Table 4). Adults had larger amounts of hydrocarbons in their profile compared to any of the brood samples, as seen from the area of the peaks compared to that of the internal standard peak (*n*-C_18)_ in Fig. 2.

### Qualitative Differences

Of the 32 compounds identified, 27 were present in all sample categories, although only in trace amounts (below 1% on average, Table 1) in some sample categories. Five compounds (3-MeC_23_, C_24:1_, C_26:1_, 11-MeC_29_ and 5-MeC_29_) were absent in developing gynes, and none of these were present in high abundance in any of the other sample categories (Table 2, Fig. 2). The cuticular profile of adult workers comprised eight compounds (four *n-*alkanes, and four alkenes) with > 1% representation on average, whereas worker pupae (cocoons and developing individuals) had the most diverse profile, with 18 compounds that comprised on average > 1% of the peak area (Table 2, Fig. 2). Seven of these were short-chained branched alkenes (five C_16_-C_19_-dimethyls, one C_20_-dimethyl, and one C_23_-dimethyl), and two were *n-*alkanes (one linear C_24_, and one branched x-MeC_21_), all of which were present only in low amounts in the other sample categories (Table 2; Fig. 2c). In the sexual brood, developing males had eight, and gynes seven compounds with > 1% average representation, and the cocoons of both had 10 compounds (Figs. 2a & 2b).

Notably, developing gynes and males carried moderate quantities (4–5%) of mono-methylated alkanes (Fig. 1). Conversely, cocoons of all sample sets carried significant quantities of alkenes, whereas developing individuals, especially males and gynes, only carried trace quantities of these. In adult workers, alkenes formed the dominant fraction of the profile, whereas mono-methylated compounds were present only in trace quantities (Fig. 1). This suggests, that the surface chemistry of cocoons is closer to that of adult workers, than that of the developing individuals – a pattern which is also visible based on the eigenvectors of the PCA (Fig. S2g in Supplementary Material). Both worker cocoons and developing worker individuals carried significant quantities of dimethyl-alkanes, which were present only in low quantities in the other sample sets.

Four *n-*alkanes (*n-*C_23_, *n-*C_25_, *n-*C_27_, *n-*C_29_) represented on average > 1% of the profile in all sample categories. The alkane *n-*C_29_ represented on average 20–45% of the profile in all brood (cocoons and developing individuals), but only about 4% of the cuticular profile of adult workers. Conversely, *n-*C_25_ encompassed a twofold fraction of the profile in adult workers, compared to brood. Furthermore, the amount of the linear alkane *n-*C_27_ was twofold in developing individuals, compared to cocoons, whereas the opposite was true for *n-*C_29_. Two alkenes (C_25:1_ and C_27:1_) dominated the profiles of worker cocoons and adult workers. These, and a third alkene (C_29:1_), also represented on average > 1% of the cuticular profiles of sexual cocoons, but not in developing individuals (Table 2; Fig. 2a, b). The cuticular profile of both sexual and worker brood also included five compounds with on average > 1% representation (*n-*C_26_ and 7-MeC_29_ in developing individuals, C_31:1_ in cocoons, and *n-*C_28_ and *n-*C_31_ in both cocoons and developing individuals), which were only present in trace amounts in adult workers. Of these, *n-*C_31_ reached ~ 5% representation in sexual cocoons, whereas *n-*C_28_ reached ~ 3% representation in developing sexual individuals.

## Discussion

The chemical profiles of the seven sets of samples: adult workers, cocoons (gynes, males and workers), and developing individuals (gynes, males and workers), differed both with respect to the classes of hydrocarbons, and the combination of compounds that dominated the profiles. We found consistently significant differences in the surface chemistry between the castes (sexuals *vs.* workers), between cocoons and developing individuals, and between developmental stages (adults *vs.* brood), except for gynes and males. The chemical profiles of adult workers were simple, dominated by eight compounds: four *n-*alkanes and their alkene counterparts. Nineteen of the 35 colonies included in this study were also used in the earlier study by Martin et al. (2013), and although we were not able to determine the precise location or nature of the double bonds in the alkenes, the chemical profiles found here were congruent with earlier studies of adults of *F. exsecta* (Martin et al. 2008c, 2012a, 2013; Martin and Drijfhout 2009b). This supports the earlier finding by Martin et al. (2012a), that colony-specific proportions of alkenes are stable across several years (c.f. Supplementary Fig. S3).

All sample sets carried colony information, irrespective of whether the dataset contained all compounds, only alkenes, or all except alkenes. However, earlier behavioral studies have shown that only (Z)-9-alkenes elicit aggressive responses towards non-nest-mates in adult workers of *F. exsecta* (Martin et al. 2008a), and thus are principally responsible for nest-mate recognition in this species (Martin et al. 2008a, 2013; Martin and Drijfhout 2009b). Hence, although the other classes of hydrocarbons do vary with colony, the ants do not appear to use this information for nestmate recognition. The variation in these classes of hydrocarbons may be attributable to genetic differences (van Zweden et al. 2009), or differences in habitat or food consumed (Liang and Silverman 2000; Mothapo and Wossler 2016).

Overall, adult workers had larger quantities of hydrocarbons than the brood, which may be attributable to the fact that the cuticle of adults is fully sclerotized and pigmented, thus providing water proofing, which is one of the main tasks carried out by cuticular hydrocarbons (Gibbs 1998). This difference notwithstanding, the same compounds were present in all sample groups (except five compounds in gynes), albeit in considerably different ratios across the sample sets. The only sample sets that did not clearly differ in their chemical profile, and in which the proportion of incorrectly classified samples was higher than in the remaining samples, were the male and gyne pupae (cocoons and developing individuals alike). This is in accordance with earlier results on adult sexuals in this species (Martin et al. 2014), other ant species (Chernenko et al. 2012, and references therein), and other social insects (Cervo et al. 2008; Nonacs and Carlin 1990) (Table S2 in Suuplementary Material).

We found that worker pupae have more complex hydrocarbon profiles than adult workers. This is in apparent contrast to earlier studies on several genera of ants, which showed that brood profiles are simpler (Fouks et al. 2011; Richard et al. 2007; Viana et al. 2001), or at best match the adult profiles (Akino et al. 1999; Bagnères et al. 1991; Elmes et al. 2002; Helanterä and d’Ettorre 2014; Souza et al. 2006). The difference was due to shorter-chained compounds that were present in pupae, but not detected in adult workers. The possible role of these compounds remains unclear. Sexual and worker pupae (cocoons, and developing individuals) had significantly higher ratios of *n-*alkanes than adult workers, and correspondingly smaller proportions of alkenes. This dominance by *n-*alkanes may follow from an absence of a synthesis of alkenes in brood. The *n-*alkanes mainly comprised long-chain *n-*alkanes, in particular C_27_ and C_29_, which were present in lower proportions in adult workers. The reason for this *n*-alkane dominance remains unclear, as pupae rarely leave the nest unless carried by workers for short distances, and would in theory have less need for waterproofing. One possible explanation is that the *n*-alkanes are used for brood recognition, which requires additional experimental studies.

We furthermore found considerable differences in the chemical profiles between cocoons and the developing individuals within them. Most alkenes were present in moderate proportions on the cocoons of all samples, as well as in adult workers, but were found only in trace quantities (< 1%) in developing individuals. Conversely, developing individuals carried moderate quantities of two compounds, the monomethyl 7-MeC_29_, and the alkane *n-*C_26_, both of which were present only in trace amounts (< 1%) in the remaining sample sets. The alkenes present on the cocoons may have been acquired from adult individuals, and/or the surrounding nest-material, rather than synthesized by the pupae themselves (Bos et al. 2011; Katzav-Gozansky et al. 2004). Although the role of alkenes in brood remains to be tested, and their precise identity determined, they may contribute to nest-mate recognition of brood, given their significant role in nest-mate recognition in adult individuals. In contrast, the compounds differentiating the developing individuals from cocoons and adults, 7-MeC_29_ and *n-*C_26_, were likely synthesized by the developing individuals, rather than acquired from the environment or from other ants, as the cocoon prevents exchange of surface hydrocarbons through physical contact with the surroundings (Bos et al. 2011; Boulay et al. 2000; Katzav-Gozansky et al. 2004; Leboeuf et al. 2016; Soroker and Hefetz 2000). The precise role of these compounds remains unknown, but they may originate from the larval stage. Indeed, the monomethyl 7-MeC_29_, and other monomethylated-C_29_ hydrocarbons, were present on larvae as well (Peignier et al. 2019)(Table S2 in Supplementary Material). These were also found on newly emerged sexuals of *F. exsecta*, but not on mature ones (Martin et al. 2014)(Table S2 in Suuplementary Material). Likewise, the longer chain-length compounds (*n-*C_31_, and C_31:1_) found in brood samples, were found on eggs (C_31:1_, Helanterä and d’Ettorre 2014), and larvae (*n-*C_31_, and C_31:1_, Peignier et al. 2019) of *F. exsecta,* but were not consistently present on adult workers, or on newly emerged or mature sexuals of this species (Martin et al. 2014) (Table S2 in Supplementary Material). Brood-specificity of these hydrocarbons potentially points towards a role as recognition cues, but they could also be involved in other brood-specific signaling, thus a more detailed investigation would be needed to be able to determine their role.

The profiles of worker brood (both cocoons and developing individuals) included a substantial fraction of dimethyl-alkanes. This sets the profiles of worker brood apart from the other sample sets, in which these compounds were found only in low quantities (Table 1). Several of these were short-chained (C_17_-C_23_) dimethyl alkanes with just over 1% representation. Short-chained hydrocarbons are relatively more volatile, which may make them less suitable for recognition purposes (Blomquist 2010). Nonetheless, also volatile chemicals have been suggested to be involved in nest-mate recognition in ants (Katzav-Gozansky et al. 2004), and short chained hydrocarbons have been shown to affect recognition in honey bees (Breed and Stiller 1992). The precise role of these compounds remains to be clarified, but these may provide chemical cues to allow discrimination between worker and sexual brood, in addition to the size of the brood items.

In an earlier study we found that adult workers treat worker and sexual pupae differently in brood recognition experiments, such that they discriminate against hetero-colonial sexual brood, but not against worker brood (Pulliainen et al. 2018). This begs the question, whether some of the compounds identified in this study may carry information on brood type (worker versus sexual), or gender, and whether workers use such information, besides potentially using the size difference, as a cue (Brian 1975; Trible and Kronauer 2017). Worker-destined larvae can be distinguished from queen-destined larvae chemically, based on the proportion of short-chained compounds—the so called ‘princess pheromone’—in *Harpegnathos* ants (Penick and Liebig 2017), and possibly by a chemical signal in *Myrmica* ants (Brian 1975). Nonetheless, evidence for recognition of the caste of brood in social insects is scarce (Achenbach et al. 2010; Villalta et al. 2016). Suitable candidates for such cues would most likely be on the cocoon, rather than the developing individual, unless the chemical cues can be perceived through the cocoon. Indeed, hydrocarbons may be perceived at short distances without necessarily involving antennal contact (Brandstaetter et al. 2008). Our study indeed found differences in short-chain dimethyl hydrocarbons between workers and sexuals, which could be used as cues for discriminating sexual brood from worker brood. However, the function of these cues as potential signal remains to be investigated in more detail.

In this study we have demonstrated clear differences in the surface chemistry among castes, and between cocoons and the developing individuals inside the cocoon. Alkenes, of which (Z)-9-alkenes have been shown to function as nest-mate recognition signals in *Formica exsecta* (Martin et al. 2008a), were present only in minimal quantities on developing individuals, but were abundant on adult workers and cocoons. Thus, our results support both the notion that nest-mate recognition cues can be acquired from the surrounding individuals and the environment (Bos et al. 2011; Boulay et al. 2000; Katzav-Gozansky et al. 2004; Leboeuf et al. 2016; Soroker and Hefetz 2000), and that the individuals acquire their colony *Gestalt* odor—a shared colony odor, sensu the *Gestalt* model defined by Crozier and Dix (1979)—during a chemical integration period at the early adult stage (Lenoir et al. 1999). These findings highlight the diversity of surface chemistry in social insects across developmental stages and suggest new avenues of exploration in the field of chemical ecology.

## Supplementary Information

Below is the link to the electronic supplementary material.

Supplementary file1 (DOCX 965 KB)

## Data Availability

DRYAD 10.5061/dryad.7pvmcvds3.
