# Supplementary material for: The Scent of Ant Brood: Caste Differences in Surface Hydrocarbons of Formica exsecta Pupae
Source: J Chem Ecol. 2021 Apr 26;47(6):513–24. doi: 10.1007/s10886-021-01275-w (PMC8217064; doi:10.1007/s10886-021-01275-w)
Supplement: Supplementary file 1 — Supplementary file1 (DOCX 965 KB) [file 10886_2021_1275_MOESM1_ESM.docx]

# Supplementary Material

# The Scent of Ant Brood: Caste Differences in Surface Hydrocarbons of *Formica exsecta* Pupae

## Unni Pulliainen^1,2^ Nick Bos^3^ Patrizia d’Ettorre^4^ Liselotte Sundström^1^*

^1^Organismal and Evolutionary Biology Research Programme, Faculty of Biological and Environmental Sciences, University of Helsinki, Finland

^2^Tväminne Zoological Station, Faculty of Biological and Environmental Sciences, University of Helsinki, Helsinki, Finland

^3^Department of Biology, Faculty of Sciences, University of Copenhagen, Denmark

^4^Laboratory of Experimental and Comparative Ethology, University of Paris 13, Sorbonne Paris Cité, Paris, France

Corresponding author: Liselotte Sundström – liselotte.sundstrom@helsinki.fi

**Table S1.** Retention times, Retention Indices, and diagnostic ions for identification of the compounds listed in Table 2 (modified and augmented from Pulliainen et al, (2018))

| Compound | Retention Time (min) | Retention Index | Diagnostic ions (*m/z*) |
| --- | --- | --- | --- |
| 4,6-diMeC16+ 4,9-diMeC16 | 6.34 | unresolved | 71/211, 112/169, 126/155; 239 |
| 8-MeC_17_ | 7.36 | 1712 | 127, 155; 239 |
| 5,7-diMeC_17_ +5,9-diMeC_17_ | 7.66 | 1754 | 84/211, 126/168, 140/155; 253 |
| 5,9-diMeC_19_ | 9.15 | 2027 | 84, 239, 155, 169; 281 |
| x,y-diMeC_19_ | 9.58 | 2068 | 281 |
| C_21_ | 11.10 | 2100 | 296 |
| 10-MeC_21_ | 11.62 | 2141 | 155, 183; 295 |
| x-MeC_21_ | 12.16 | 2183 | 295 |
| C_23:1_ | 13.53 | 2274 | 322 |
| C_23_ | 13.87 | 2300 | 324 |
| 3-MeC_23_ | 15.04 | 2376 | 57, 309; 281; 323 |
| C_24:1_ | 15.04 | 2376 | 336 |
| 3,11-DiMeC_23_ | 15.32 | 2394 | 57, 323, 196, 183; 337 |
| C_24_ | 15.41 | 2400 | 338 |
| C_25:1_ | 16.61 | 2476 | 350 |
| C_25_ | 17.00 | 2500 | 352 |
| 3-MeC_25_ | 18.18 | 2572 | 57, 337, 309; 351 |
| C_26:1_ | 18.21 | 2574 | 364 |
| C_26_ | 18.63 | 2600 | 366 |
| C_27:1_ | 19.87 | 2675 | 378 |
| C_27_ | 20.28 | 2700 | 380 |
| 3-MeC_27_ | 21.44 | 2772 | 57, 365, 337; 379 |
| C_28_ | 21.89 | 2800 | 394 |
| C_29:1_ | 23.14 | 2877 | 406 |
| C_29_ | 23.52 | 2900 | 408 |
| 11-MeC_29_ | 23.90 | 2924 | 168, 281, 252; 407 |
| 9-MeC_29_ | 24.00 | 2930 | 141, 309; 407 |
| 7-MeC_29_ | 24.10 | 2937 | 112, 337, 309; 407 |
| 5-MeC_29_ | 24.30 | 2949 | 85, 365, 337; 407 |
| C_30_ | 25.10 | 3000 | 422 |
| C_31:1_ | 26.31 | 3079 | 434 |
| C_31_ | 26.63 | 3100 | 436 |

**Table S2** Heat map of compounds with on average >1% representation in at least one of the developmental stages (eggs, larvae, pupae, and adults) of *Formica exsecta.* Data from eggs (Helanterä & d’Ettorre 2014), larvae (Peignier et al. 2019), pupae, adult workers (this study), and emerged and mature sexuals (Martin et al. 2014). The percentages of compounds in emerged and mature sexuals were estimated from Fig. 1 in Martin et al. (2014).

|  |  |  | | Pupae | | | | | | | | | Adults | | | | | | |
| --- | --- | --- | --- | --- | --- | --- | --- | --- | --- | --- | --- | --- | --- | --- | --- | --- | --- | --- | --- |
|  |  |  | | Workers | | Gynes | | | | Males | | | Workers | Gynes | | | Males | | |
| Compound | Eggs | Larvae | | Cocoon | Individual | Cocoon | Individual | | | Cocoon | | Individual | Adult | Emerged | Mature | | | Emerged | Mature |
| 4,6-diMeC16 + 4,9-diMeC16 |  |  | |  |  |  |  | | |  | |  |  |  |  | | |  |  |
| 8-MeC17 |  |  | |  |  |  |  | | |  | |  |  |  |  | | |  |  |
| 5,7-diMeC17 +5,9-diMeC17 |  |  | |  |  |  |  | | |  | |  |  |  |  | | |  |  |
| 5,9-diMeC19 |  |  | |  |  |  |  | | |  | |  |  |  | 1--5% | | | |  |
| x,y-diMeC20 |  |  | |  |  |  |  | | |  | |  |  |  | **5--10%** | | | |  |
| C_21:1_ |  |  | |  |  |  |  | | |  | |  |  |  | **10--20 %** | | | |  |
| C_21_ |  |  | |  |  |  |  | | |  | |  |  |  | **> 20 %** | | | |  |
| 10-MeC21 |  | |  |  |  |  | |  |  | |  | |  |  |  |  | | |  |
| x-MeC_21_ |  | |  |  |  |  | |  |  | |  | |  |  |  |  | | |  |
| C_23:1_ |  | |  |  |  |  | |  |  | |  | |  |  |  |  | | |  |
| C_23_ |  | |  |  |  |  | |  |  | |  | |  |  |  |  | | |  |
| 3,11dimeC23 |  | |  |  |  |  | |  |  | |  | |  |  |  |  | | |  |
| C_24_ |  | |  |  |  |  | |  |  | |  | |  |  |  |  | | |  |
| C_25:1_ |  | |  |  |  |  | |  |  | |  | |  |  |  |  | | |  |
| C_25_ |  | |  |  |  |  | |  |  | |  | |  |  |  |  | | |  |
| x-MeC_25_ |  | |  |  |  |  | |  |  | |  | |  |  |  |  | | |  |
| C |  | |  |  |  |  | |  |  | |  | |  |  |  |  | | |  |
| D |  | |  |  |  |  | |  |  | |  | |  |  |  |  | | |  |
| C_26_ |  | |  |  |  |  | |  |  | |  | |  |  |  |  | | |  |
| C_27:1_ |  | |  |  |  |  | |  |  | |  | |  |  |  |  | | |  |
| C_27_ |  | |  |  |  |  | |  |  | |  | |  |  |  |  | | |  |
| 7-MeC_27_ |  | |  |  |  |  | |  |  | |  | |  |  |  |  | | |  |
| C_28_ |  | |  |  |  |  | |  |  | |  | |  |  |  |  | | |  |
| C_29:1_ |  | |  |  |  |  | |  |  | |  | |  |  |  |  | | |  |
| C_29_ |  | |  |  |  |  | |  |  | |  | |  |  |  |  | | |  |
| 11-MeC_29_ |  | |  |  |  |  | |  |  | |  | |  |  |  |  | | |  |
| 9-MeC_29_ |  | |  |  |  |  | |  |  | |  | |  |  |  |  | | |  |
| 7-MeC_29_ |  | |  |  |  |  | |  |  | |  | |  |  |  |  | | |  |
| 5-MeC_29_ |  | |  |  |  |  | |  |  | |  | |  |  |  |  | | |  |
| C_31:1_ |  | |  |  |  |  | |  |  | |  | |  |  |  |  | | |  |
| C_31_ |  | |  |  |  |  | |  |  | |  | |  |  |  |  | | |  |
| Mix x-MeC_31_ |  | |  |  |  |  | |  |  | |  | |  |  |  |  | | |  |
| C_32_ |  | |  |  |  |  | |  |  | |  | |  |  |  |  | | |  |
| C_33:2_ |  | |  |  |  |  | |  |  | |  | |  |  |  |  | | |  |
| C_33_ |  | |  |  |  |  | |  |  | |  | |  |  |  |  | | |  |
| C_35:1_ |  | |  |  |  |  | |  |  | |  | |  |  |  |  | | |  |


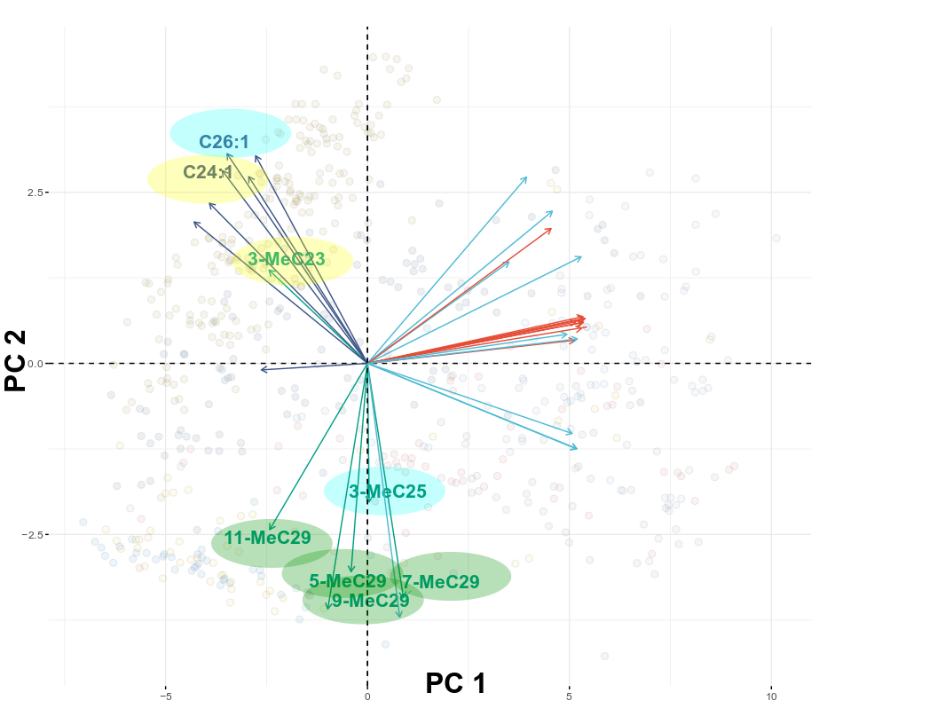

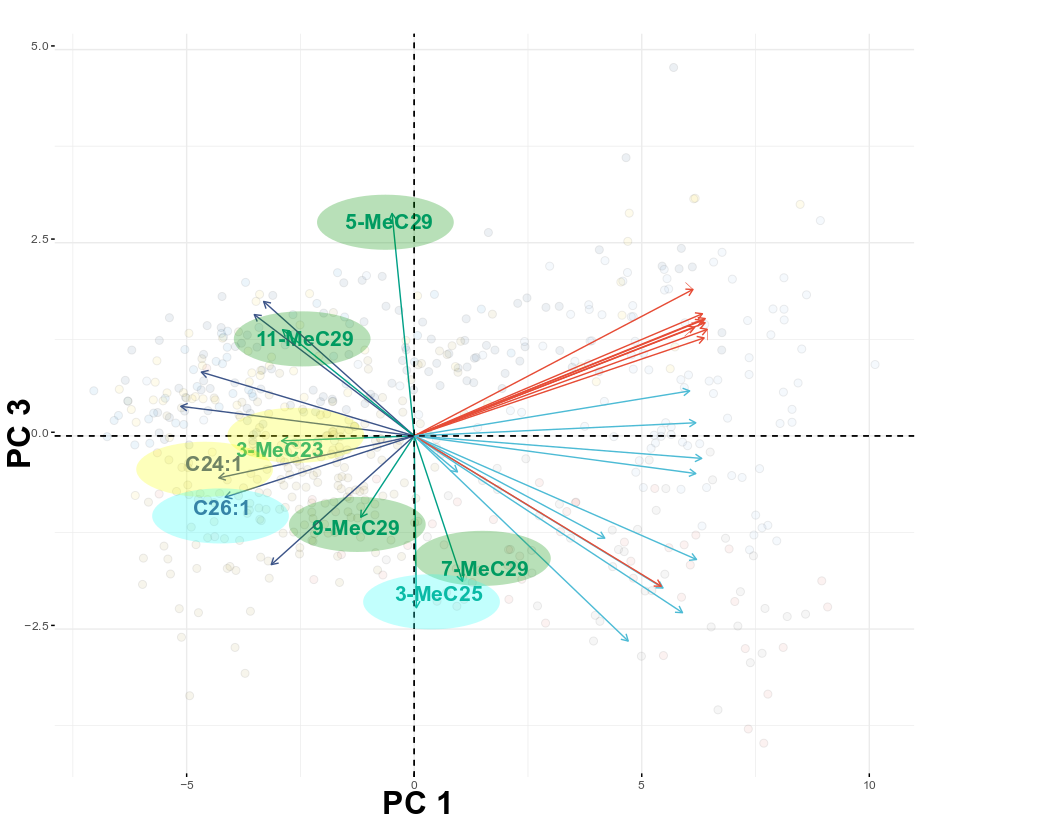


**Fig. S1** Biplots of PC1 and PC2/PC3, showing the direction in which the different hydrocarbons (arrows) contribute to these principal components. All samples are shown in the background (grey dots). The coeluted compounds C_26:1_ and 3-MeC_25_ are marked in blue, C_24:1_ and 3-meC_23_ in yellow, and 5-, 7-, 9-, and 11-MeC_29_ in green. Figure (a) shows how coeluted C_26:1_ and 3-MeC_25_ contribute in opposite directions on PC2, and figure (b) shows how 7-MeC_29_ and 9-MeC_29_ contribute in the opposite direction to 5-MeC_29_ and 11-MeC_29_ on PC3


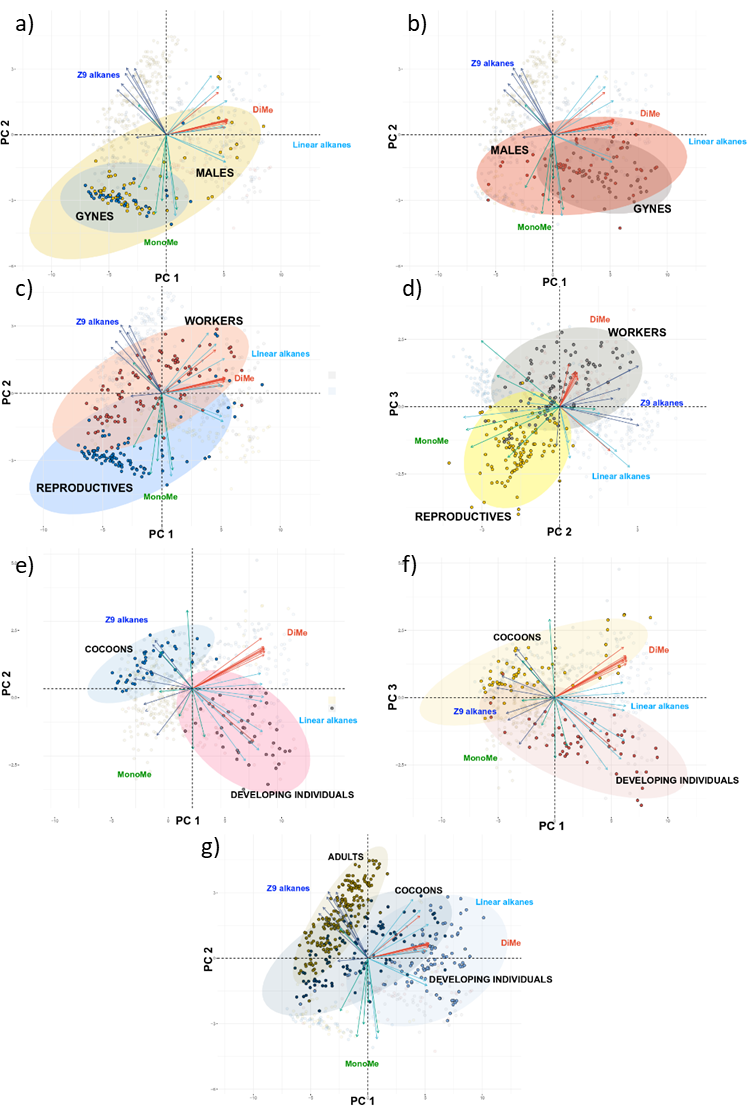


**Fig. S2** Biplot of the two principal components (PCs) that best separated the samples (coloured dots) in each comparison: a) cocoons of males and gynes b) developing males and gynes c) cocoons of reproductives and workers d) developing reproductives and workers e) gyne cocoons and developing individuals f) male cocoons and developing individuals g) worker cocoons, developing individuals and adults. Arrows show the direction in which different compounds contribute to these principal component dimensions, with different classes of hydrocarbons (alkenes, linear alkanes, dimethylated and monomethylated alkanes) shown with arrows of different colours.


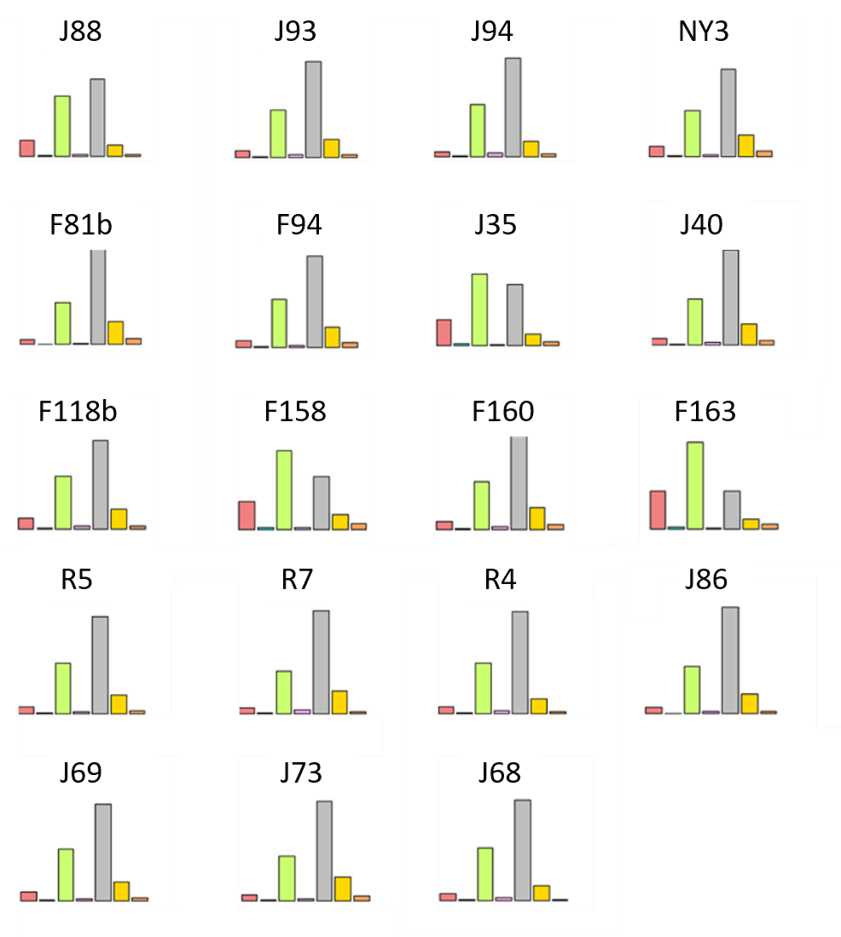


**Fig. S3** Of the 35 colonies of *F. exsecta* sampled in this study, 19 were also included in the Martin et al. (2013) study. The proportions of alkenes in adult workers (colony code above each plot) closely match those found for the same colonies in Martin et al. (2013, Figure 1). Red bars = C_23:1_, blue = C_24:1_, green = C_25:1_, pink = C_26:1_, grey = C_27:1_, orange = C_29:1_, brown = C_31:1_.
